# Biological Reconstruction Following the Resection of Malignant Bone Tumors of the Pelvis

**DOI:** 10.1155/2013/745360

**Published:** 2013-04-03

**Authors:** Frank Traub, Dimosthenis Andreou, Maya Niethard, Carmen Tiedke, Mathias Werner, Per-Ulf Tunn

**Affiliations:** ^1^Department of Orthopedic Oncology, Sarcoma Center Berlin-Brandenburg, HELIOS Klinikum Berlin-Buch, Schwanebecker Chaussee 50, 13125 Berlin, Germany; ^2^Departement of Pathology, Sarcoma Center Berlin-Brandenburg, HELIOS Klinikum Emil von Behring, Walterhöferstr. 11, 14165 Berlin, Germany

## Abstract

*Background*. Surgical treatment of malignant pelvic bone tumors can be very challenging. The objective of this retrospective study was to evaluate the oncological as well as the clinical and functional outcome after limb salvage surgery and biological reconstruction. *Methods*. The files of 27 patients with malignant pelvic bone tumors, who underwent surgical resection at our department between 2000 and 2011, were retrospectively analyzed (9 Ewing's sarcoma, 8 chondrosarcoma, 4 osteosarcoma, 1 synovial sarcoma, 1 malignant fibrous histiocytoma, and 4 carcinoma metastases). *Results*. After internal hemipelvectomy reconstruction was performed by hip transposition (*n* = 16), using autologous nonvascularised fibular graft (*n* = 5) or autologous iliac crest bone graft (*n* = 2). In one patient a proximal femor prothetis and in three patients a total hip prosthesis was implanted at the time of resection. The median follow-up was 33 months. Two- and five-year disease-specific survival rates of all patients were 86.1% and 57.7%, respectively. The mean functional MSTS score was 16.5 (~55%) for all patients. *Conclusion*. On the basis of the oncological as well as the clinical and functional outcome, biological reconstruction after internal hemipelvectomy seems to be a reliable technique for treating patients with a malignant pelvic bone tumor.

## 1. Introduction

Chondrosarcomas, Ewing's sarcomas, and osteosarcomas are the most common primary bone sarcomas of the pelvis and account for 5% to 10% of all malignant bone tumors.[1, 2]. The prognosis and survival of patients with bone sarcomas in this location are much less favorable than for patients with tumors of the extremities. Additionally the pelvis is the second most common site of bone metastases after the spine.

**Figure 1:**
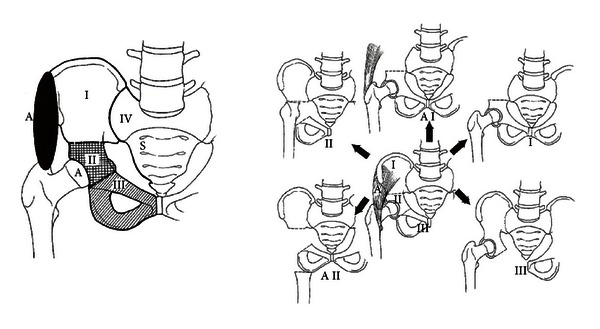
A Classification of pelvic resection [5].

The treatment of malignant bone tumors involving the pelvis is a great challenge to the orthopaedic surgeon in terms of local control owing to the complexity of pelvic anatomy, which increases the difficulty of resection and reconstruction. First attempts to excise malignant bone tumors of the pelvis were reported by Enneking in 1966 [3] and Steel in 1978 [4]. Resection of the tumor can be performed either by internal or external hemipelvectomy. Pelvic resections have been classified by the Musculoskeletal Tumor Society into 4 resection types: type I (iliac), type II (periacetabular), type III (os pubis, ischii), and type IV (sacrum) [5–8]; see also Figure 1.

Because of the improvements in imaging modalities and in multimodal treatment plans, leading to a prolonged patient survival, limb sparing procedures are usually the treatment of choice, especially considering the low patient acceptance of hindquarter amputation.

The reconstruction procedures after internal hemipelvectomy include endoprosthetic replacement [9] and biological reconstruction using autografts or allografts [10–12] as well as hip transposition [13].

The aim of this report was to evaluate patients with malignant tumors of the pelvis after biological reconstruction with regard to oncological, clinical, and functional outcomes.

## 2. Material and Methods

The medical files of 27 patients with a malignant pelvic bone tumor surgically treated at our institution between 2000 and 2012 were retrospectively analyzed. All patients had signed a consent form at hospital admission, allowing the use of anonymized information for research purposes.

There were 12 female and 15 male patients with an average age of 44.6 years (range 10–77 years) at the time of the first surgical intervention. According to the histological report, the primary tumor was recorded as Ewing's sarcoma in 9 patients, chondrosarcoma in 8, osteosarcoma in 4, synovial sarcoma, and malignant fibrous histiocytoma of the bone in one patient each, respectively. Four patients presented with solitary metastases to the pelvis from renal cell carcinoma in two cases, thyroid cancer in one and invasive ductal carcinoma of the breast in another patient. Tumor volume was assessed by the pathologist during examination of the surgical specimen or in the Ewing's sarcoma by the radiologist before neoadjuvant treatment was started. The average tumor volume was 451 cm³ (214–2200 cm³).

All patients diagnosed with an osteo- or Ewing's sarcoma received neoadjuvant chemotherapy as determined by the appropriate protocols. One patient with a Ewing's sarcoma received a combination of radiation and chemotherapy prior to surgery.

**Figure 2:**
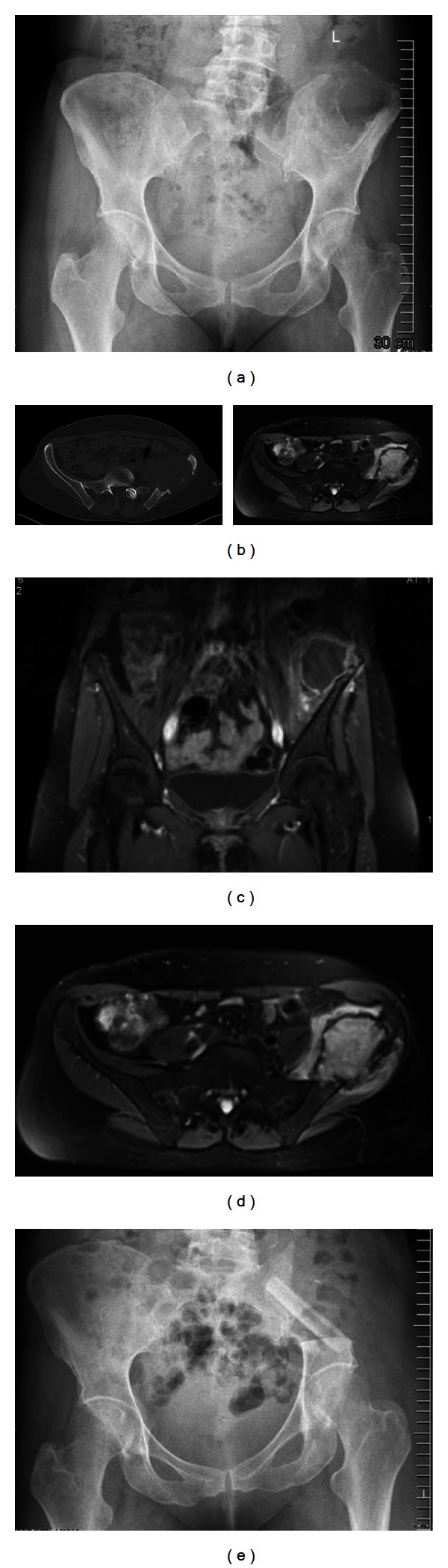
(a) Anteroposterior radiograph of the pelvis, showing a large osteolytic lesion of the left iliac bone (synovial sarcoma). (b) CT scan of the same patient showing the size of the tumor. Notably is the lack of matrix or calcification inside the tumor. (c) and (d) MRI scan of the same patient showing the intra- and extrapelvine size. (e) Postoperative X-ray after P1 resection and pelvic reconstruction stabilised with an autologous nonvascularised fibular graft.

**Figure 3:**
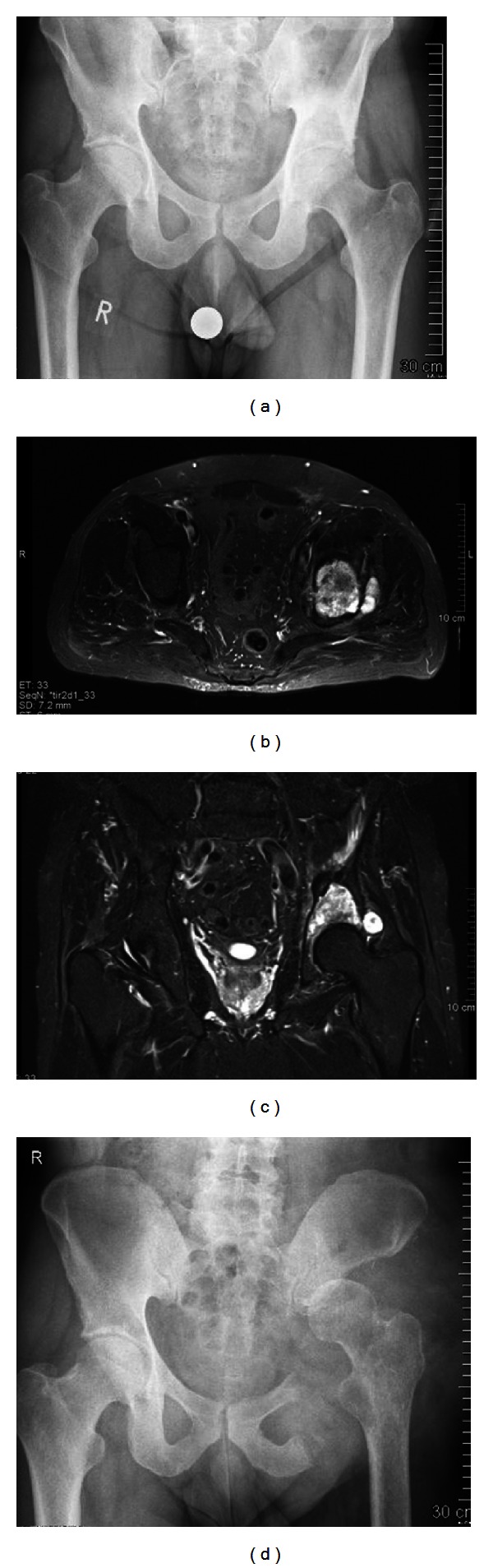
(a) Anteroposterior radiograph of the pelvis, showing a periacetabular chondrosarcoma on the left. (b) and (c) MRI of the pelvis, showing the destruction of the cortical bone and extraosseous tumor expansion. Notably is that the hip joint is not infiltrated. (d) Anteroposterior radiograph after P2 resection and hip transposition.

Sixteen patients had a hip transposition after a resection involving the acetabulum. This procedure was first described by Gebert et al. [13], the procedure involved moving the femoral head proximally to the lateral side of the sacrum or the underside of the resected ilium after resection of the acetabulum (Figure 3). The joint capsule was reconstructed with use of a polyethylene terephthalate mesh tube (Implantcast, Buxtehude, Germany), which was fixed to the pelvis with transosseous sutures and formed a pouch for the femoral head. Soft tissues were reattached to the tube. Five patients had a P1 resection and pelvic reconstruction stabilized with an autologous nonvascularized fibular graft, and in two patients an autologous iliac crest bone graft was used for the pelvic reconstruction after P1 resection (Figure 2). In one patient an endoprosthetic replacement of the hip was already done before the diagnosis of the pelvic tumor, and in three patients the resection of the femoral head was required to achieve wide surgical margins. In these three cases a femoral respectively a total hip prosthesis was implanted at the time of resection.

Surgical margins were divided into intralesional, marginal, wide, and radical, according to the classification of Enneking et al. [14]. The Musculoskeletal Tumor Society (MSTS) scoring system for the lower limb was employed to assess the functional outcome [15].

A major complication was defined as one that necessitated additional surgical intervention. A minor complication was defined as one that necessitated nonoperative management.

Survival analysis was performed using the Kaplan-Meier method. Disease-specific survival was calculated from the date of diagnosis (biopsy) until death related to disease or treatment and event-free survival from the date of tumor resection until disease recurrence or death (Figure 5).

## 3. Results

The characteristics of the patients are summarized in Table 1. The average blood loss was 2050 mL (range 900 mL–3100 mL). Bed rest was normally seven days. In eight patients it was extended to 10–14 days. Patients stayed in the hospital an average of 27.7 days after surgery (range 15–69 days). At the time of discharge all patients were able to walk using crutches or a walking frame.

At the time of the last follow-up 15 patients were alive with no evidence of disease, 5 patients were alive with disease, and 7 patients had died from disease. The median follow-up was 33 months. Two- and five-year disease-specific survival rates of all patients were 86.1% and 57.7%, respectively. Surgical margins were classified as wide in 20 patients. In four patients marginal resection were achieved, and three patients had an intralesional resection. Two patients experienced a local relapse (one osteosarcoma and one Ewing's sarcoma), although the surgical margins were wide. Both patients received a second-line chemotherapy and palliative irradiation in the further course of the disease. The two patients died of isease 29 respectively 51 month after primary diagnosis. Five patients with a primary bone tumor and one patient with metastatic renal cell carcinoma died from metastatic disease without local recurrence after an average of 32 month after diagnosis of the pelvic tumor.

**Figure 4:**
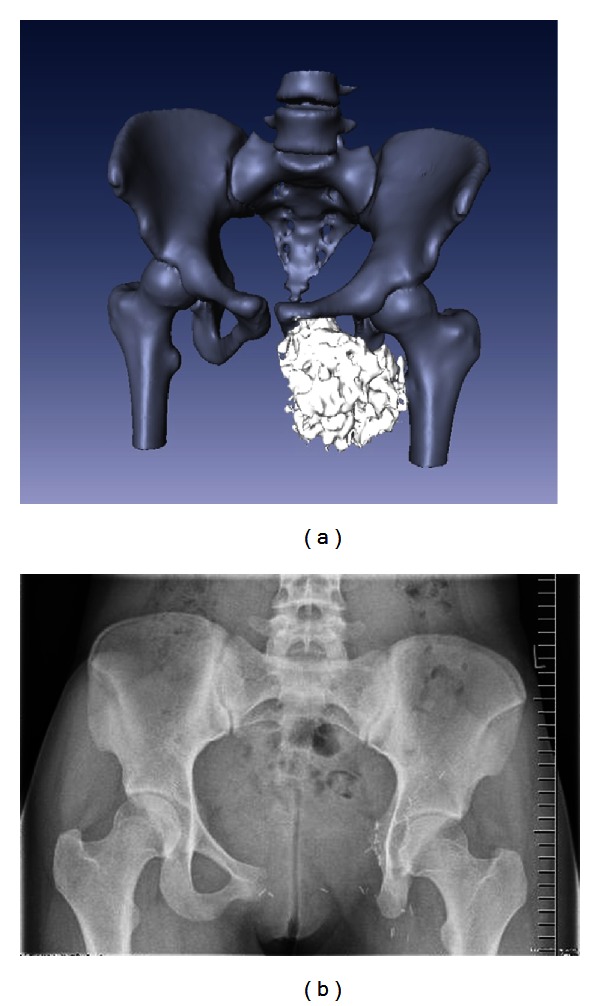
(a) CT reconstruction of the pelvis of a 15-year-old girl with a chondrosarcoma of the left os pubis and os ischii. (b) Anteroposterior radiograph after P3 resection.

**Figure 5:**
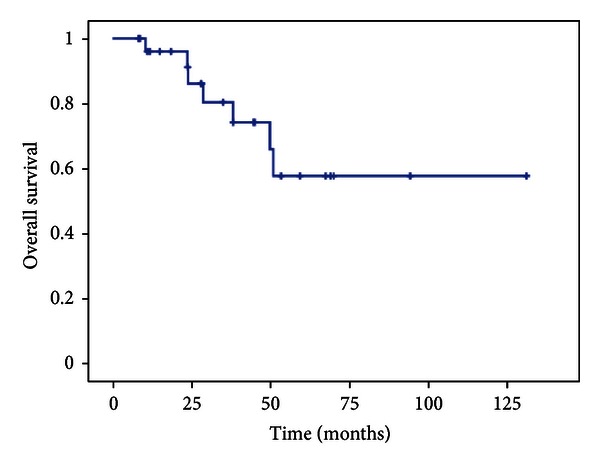
Kaplan-Meier plot showing the overall survival of all patients.

The mean functional MSTS score was 16.5 (~55%) for all patients. Three patients were able to walk without any support (Figure 4), two had a transposition after P2-3 resection, and the other patient had a P1 resection and was reconstructed with an autologous iliac crest bone graft. All the other patients need at least one cane for longer distances. The MSTS score in the subgroups after resection and biological reconstruction was after P1 resection 16,9 (10–26), after P1-2 resection 16 (14–18) and after P1-3 resp. P2-3 resection 17,4 (9–30). The MSTS score in the patient with the P1+4 resection was 18, and in the patient after P2-4 resection was 20.

There were nine complications which required an operative intervention. Four patients developed a superficial postoperative wound infections involving the skin. All healed after revision surgery. In one patient a previously implanted Hickman line had to be changed short time after the surgery, because of sepsis. In two patients the endoprosthesis had to be removed because of dislocation and septic loosening. In one patient with a fibular autograft after P1 resection there was an osteomyelitis of the bone graft, and a sequestrum had to be removed. Shortly after this procedure a postoperative pseudarthrosis was observed, but causing no problems. And in one patient a paresis of the leg developed directly after the surgery, because the sciatic nerve had to be resected.

## 4. Discussion

In the operative treatment of malignant tumors in the pelvis, limb-salvage surgery, combined with chemo- or radiotherapy, showed similar survival, recurrence, and complication rates as well as an improvement in the quality of life of the patients when compared to hindquarter amputation [16, 17]. The overall survival of patients with a pelvic sarcoma is often far worse than for those with one in an extremity [18, 19]. This poor prognosis may be partially attributable to the fact that pelvic sarcomas are often diagnosed in an advanced stage, when the tumor is more likely to be large in size [2, 17]. As studies have shown that limb-salvage techniques and the amputation show no difference in terms of the survival rate of patients with malignant bone tumors, the limb-salvage techniques are now being frequently used even for cases of advanced tumors. Tumor size and localization are the determining factors when it comes to decide which reconstruction technique is employed following limb sparing surgery. The bony defect in type I resections can be reconstructed with autograft fibula, cortical or pelvic allograft, or bone cement. The advantages of replacing the resected bone are pelvic stability and maintenance of limb length. No formal reconstruction is required for type III resections [16]. The hip transposition technique involves refixation of the inferior part of the acetabulum to the preserved bone into an artificial capsule that is attached to the intact proximal bone (ilium or sacrum).

In our series, acceptable functional results, with an average MSTS score of 16 could be achieved after a median of 33 months prospective followup examination. Thus our results are comparable to the findings in the literature [9, 10, 20]. Compared with MSTS scores after hemipelvic endoprosthesis reconstruction our results are equal [9, 21]. Because of the fact that hemipelvic megaprosthetic replacement is associated with a high complication rate and the fixation of the megaprosthesis in the pelvic bone as well as loosening of the prosthesis are still major problems, we recommend the biological reconstruction using hip transposition [13] or reconstruction of the pelvic stability by bone autografts [10].

The indications for pelvic reconstruction include young patients, resection of weight-bearing or -moving elements (such as the hip joint), primary sarcomas, and solitary pelvic bone metastasis in patients with “favorable” cancers such as thyroid, renal, and breast cancer with long life expectancies [22]. From the oncological point of view the outcome of the patients with a primary pelvic tumor should be differentiated from that of patients with a metastasis. In our study the survival did not differ significantly. The rate of metastasis in our study is similar to the one reported by other authors, potentially reflecting more biological aspect of the disease than the operative approach [2, 10, 23]. When the lesion is small but causes destruction of the hip joint, a hip replacement can be performed. However, implant stability may be impaired by the cancer and/or any postoperative chemotherapy or radiation therapy. When cancer has destroyed the acetabulum to the extent that it is no longer a contained defect, more extensive surgical procedures are necessary. In these cases, en bloc resection of the diseased bone is performed, using the same surgical principles to achieve tumor-free margins of resection as for primary bone tumors, and perform hemipelvectomy [23, 24]. Although these procedures are associated with increased morbidity and mortality rates that require longer hospitalization and rehabilitation [21, 25], we consider this approach for appropriate when locally advanced disease precludes internal stabilization. Limited data are available regarding the survival of patients with solitary pelvic metastases [24, 25]. Patients with solitary pelvic metastases seem to have favorable survival times, thus we think this may justify consideration of a radical surgical approach. However, it is not proven that major surgeries are related with an improved survival compared to curettage in patients with pelvic metastases [26].

## 5. Conclusion

The use of limb-salvage pelvic resections has increased with the advances in imaging and surgical techniques and instrumentation. However, pelvic surgery for malignant bone tumors remains challenging because of the complex anatomy and the extent of tumor growth.

## Figures and Tables

**Table 1:** 

	*n*
Patients	27
Female 12, male 15	
Age	44.6
	(10,3–77,2)
Diagnosis	
Ewing's sarcoma	9
Chondrosarcoma	8
Osteosarcoma	4
Synovial sarcoma	1
Malignant fibrous histiocytoma	1
Metastasis-renal cell carcinoma	2
Metastasis-invasive ductal carcinoma of the breast	1
Metastasis-thyroid cancer	1
Tumor stage (Enneking)	
Ib	1
IIa	2
IIb	20
IV (metastasis)	4
Grading (for primary tumors)	
G1	1
G2	5
G3	17
Neoadjuvant therapy	
Polychemotherapy	15
Radiotherapy + polychemotherapy	1
Adjuvant therapy	
Polychemotherapy	8
Radiotherapy	3
Radiotherapy + polychemotherapy	4
Resection type (according to Enneking)	
P1	6
P1-2	3
P1-3	5
P2-3	11
P2-4	1
P1+4	1
Regression after neoadjuvant treatment available for 4 osteosarcoma and 7 Ewing's sarcomas according to Salzer-Kuntschik	
Grade 1	2
Grade 3	3
Grade 4	4
Grade 5	2
Surgical margins	
Wide	20
Marginal	4
Intralesional	3
Oncological outcome	
No evidence of disease (NED)	15
Alive with disease (AWD)	5
Died of disease (DOD)	7
